# London

**Published:** 1920-09-04

**Authors:** 


### THE MEDICAL STUDENT AND HIS WORK.

### THE MEDICAL SCHOOLS OF THE UNITED KINGDOM.

### LONDON.

**University of London, University College** (Faculty of Medical Sciences) : University Centre for Preliminary and Intermediate Medical Studies.—The College Faculty of Medical Sciences comprises the departments of physics, chemistry, botany, and zoology (the preliminary medical sciences), also the departments of anatomy, physiology, and pharmacology (the intermediate medical sciences), and the department of hygiene and public health (post-graduate study). Composition Fees :. For the courses required by the University of London : (1) For the first medical course, 35 guineas, entitling to one attendance. (2) For the second medical course, 80 guineas, payable in two instalments of 40 guineas each. This fee entitles to attendance on anatomy and physiology during three years, and to one attendance on organic and applied chemistry, pharmacology and materia medica, and to the privileges of the Union Society (including the use of the gymnasium and the athletic ground at Perivale) for two sessions. For the medical education required by the Examination Board in England and the Society of Apothecaries : First examination, Parts I., II., III., 29 guineas, entitling to one attendance and to the privileges of the Union Society for one session. First examination, Part IV., and second examination, 80 guineas, payable in two instalments of 40 guineas each. This fee entitles to attendance during three years (including one attendance on each practical course in physiology) in all subjects but practical pharmacy, the fee for which is three guineas, and to the privileges of the Union Society (including the use of the gymnasium and the athletic ground at Perivale) for two sessions.

The new chemical laboratories, which occupy a large area in Gower Place on the north side of the College, and include accommodation for the teaching of chemistry to medical students, are now open.

All departments in the Faculty of Medical Sciences are open to women students on the same terms as to men.

**Charing Cross Hospital.**—This hospital is fully organised for University teaching, and contains 300 beds. The courses of study are specially designed to meet the requirements of the University of London degrees, the Conjoint Board diplomas, and the final studies of the Universities of Oxford and Cambridge. Men and women students are admitted on equal terms. Fully equipped laboratories are available for pathological and bacteriological work. Fees: Entrance fees—(1) Primary and Intermediate, 10 guineas; (2) Final, 8 guineas. Annual fee (including Club and Library subscriptions), 26 guineas. The usual resident and students' appointments are open to students, and valuable prizes are awarded annually. Full information respecting the Medical School, and the course of study recommended, may be obtained on application to the Dean, W. J. Fenton, M.D., F.R.C.P., Charing Cross Hospital Medical School, London, W.C. 2. [Telephone Number, City 8015.]

**Guy's Hospital.**—This hospital contains 644 beds, and has a large Medical School. There are numerous special departments, and a large number of student and resident appointments available. Special classes are held for the London University, Oxford, Cambridge, and Fellowship courses. Scholarships and prizes to the amount of £800 are awarded annually. For further particulars application should be made to the Dean of the Medical School.

**King's College Hospital Medical School (University of London).**—King's College Hospital is situated at Denmark Hill, London, S.E., and at present contains 400 beds. The number will be increased eventually to 600. There are about 3,500 attendances per week in the casualty and out-patient departments. The hospital possesses every modern requirement for both patients and students. The Medical School contains fine class-rooms, library, lecture theatre, pathological laboratory, dining room, common rooms, etc. In the education at the Hospital a special feature has always been the individual attention given to each student, and each subject is taught by its own specialist. There is ample opportunity for any student who wishes to specialise in a subject to continue his studies with the help of junior staff appointments. The teaching of the preliminary subjects, including chemistry, physics, biology, anatomy, and physiology, is given at King's College in the Strand, students coming to the hospital for their advanced work. Fifteen resident appointments are made half-yearly. The composition fee for advanced medical studies is 93 guineas if paid in one instalment, or 95 guineas if paid in two instalments, in addition to the entrance fee of 10 guineas. The composition fee for the whole University or Conjoint course is 200 guineas. Two Entrance Scholarships (£50 each), for University students, will be offered for competition in September 1920. Full particulars of these scholarships, and the school prospectus, may be obtained by application to either of the following : H. Willoughby Lyle, M.D., B.S. (Lond.), F.R.C.S., *Dean* ; S. C. Ranner, M.A. (Cantab.), Secretary of the Medical School, King's College Hospital, Denmark Hill, London, S.E. 5.

**London Hospital Medical College.**—The London Hospital is the largest institution of its kind in England, and contains 933 beds. Being situated in the East End, it ministers to a very large and poor district. The total number of patients during 1917 was 118,183, made up of 16,053 in-patients and 102,130 out-patients. During the year 6,656 major operations were performed. From the beginning of the war to the close of the year 1917, 4,771 military patients received treatment, and naval patients (who were first admitted at the end of September) numbered 396. Five entrance scholarships are offered annually, including the Price Scholarship in science, of £100; the Epsom Scholarship; and the entrance scholarships in science, anatomy and physiology, and arts. The annual inclusive fee is 30 guineas. Appointments: Registrars, resident accoucheurs, house physicians, house surgeons, etc. One hundred and forty-one appointments are made annually. All appointments are free to full students, and all resident appointments have board free. Full information may be obtained from the Dean of the Medical College. A Hostel for students is provided on the hospital ground. The Clubs Union Athletic Ground is within easy reach of the hospital.

**London Hospital Dental School.** — The school, which is fully equipped on the most modern lines and with the latest appliances, is an integral part of the college and hospital, and is admirably adapted for the purpose of teaching. The school possesses, in addition to the theatres, laboratories, and museums of the college, a special museum of dental anatomy and surgery, operative dentistry, prosthetic and extraction rooms, and laboratories for practical dental metallurgy, dental prosthesis, and porcelain work. A systematic course of instruction in dental prosthesis is arranged for pupils. The up-to-date laboratory contains every modern apparatus, and is in charge of a skilled curator and his assistants. Students in the school will enjoy the great advantage of pursuing the medical portion of the dental curriculum in a well-equipped and successful college and hospital, of which the Dental School is part. Lecturers: E. Sprawson, M.C., H. Chapman, W. Thew, H. D. Clapham, A. Dinnis, F. Butler, R. J. Probyn-Williams, Professor W. Wright, H Candy; Director of Dental Studies: E. Sprawson, M.C., M.R.C.S., L.R.C.P., L.D.S.; Dean: Professor Wm. Wright, M.B., D.Sc., F.R.C.S.

**London (Royal Free Hospital) School of Medicine for Women.**—The Royal Free Hospital is in Gray's Inn Road, and the London School of Medicine for Women is in Hunter Street, Brunswick Square, W.C. Under agreements the clinical practice of St. Mary's Hospital, the Elizabeth Garrett Anderson Hospital, the Cancer Hospital, the National Hospital for Nervous Diseases, and the Hospital for Sick Children are also available for students of the school. Residence is provided in the Students' Chambers attached to the school. There are numerous scholarships and prizes offered annually in connection with the school, among which may be mentioned the Isabel Thorne Scholarship of £30, the St. Dunstan's Medical Exhibition of £60 a year for three or five years, the Mabel Sharman-Crawford Scholarship of £20 a year for four years, the Mrs. George M. Smith Scholarship of £50 a year for three or five years, the Sir Owen Roberts Scholarship of £75 a year for four years, the Dr. Margaret Todd Scholarship of £37 10s. a year for four years, the Sarah Holborn Scholarship of £20 a year for three or five years, and the Agnes Guthrie Bursary for dental students of £50. Numerous resident appointments are available for students of the school after qualification. The session will begin on Friday, October 1.

**Middlesex Hospital.**—The hospital and medical school are situated in Mortimer Street, and only a few minutes' walk from Goodge Street Station (Hampstead and Charing Cross Tube), Oxford Circus Stations (Bakerloo and Central London Tubes), and Great Portland Street Station (Metropolitan Railway). The hospital contains 455 beds, including special wards for cancer cases, maternity and gynæcological cases, and for diseases of the skin and eye. The cancer wing (containing ninety-two beds) and special investigation laboratories offer unrivalled opportunities for the study of cancer, both in its clinical and pathological aspects. In the electro-therapeutic department students obtain instruction in the treatment of lupus and cancer by the $x$-ray method. An electrocardiographic department has recently been established. The teaching staff of the Medical School consists of six University Professors, twenty-nine lecturers, six demonstrators, and three tutors. The Bland-Sutton Institute of Pathology contains a lecture theatre, large laboratories for pathological, bacteriological, and clinical investigation, and smaller rooms for original research work. Special courses of instruction are arranged twice yearly for the Diploma of Public Health. The Anatomical and Pathological Museum has been rebuilt. Six house physicians, eight house surgeons, two obstetric and gynæcological surgeons, two casualty medical officers, two casualty surgical officers, one resident anæsthetist, and two resident officers to the special departments are appointed annually, without extra fee of any kind. Non-resident qualified clinical assistants are appointed to assist in the various out-patient departments. Numerous scholarships, medals, and prizes, to the value of over £1,000, are open for competition among students of the school. All students join the Amalgamated Students' Club, which includes the following: The Medical Society, the Common Room Society, the Cricket Club, the Football Clubs, the Athletic Clubs, the Rowing Club, the Musical Society, the Chess Club, the Lawn Tennis Club, and the Hockey Club. The winter session will open on Wednesday, October 1, at 3 P.M. For prospectus and full particulars application should be made to the Dean of the Medical School.

**St. Bartholomew's Hospital Medical School.**—St. Bartholomew's is the oldest and second largest hospital in London. The medical school is complete in every department, and provides lectures and practical laboratory teaching in the preliminary sciences and intermediate subjects, as well as in the purely professional and clinical parts of the curriculum. Scholarships and prizes to the total value of nearly £1,000 are awarded annually.

**St. George's Hospital.**—The winter session commences on October 1. Students can enter at any time. Special courses are given, in which the requirements of University and other examinations receive careful attention. The hospital contains 350 beds, and has a convalescent hospital of 100 beds at Wimbledon. The usual resident and students' appointments are open to students. Scholarships and endowed prizes, amounting to £576, are awarded every year. The entire teaching and laboratories are now devoted to purely clinical subjects, to the great advantage of students in their fourth and fifth years of study. Arrangements have been made with the University of London for students who enter St. George's during the first, second, or third year of the curriculum to carry out the necessary courses of instruction at either University College or King's College. Students have therefore the advantages of the lectures and practical classes of these Colleges of the University during the preliminary and intermediate portions of their studies, and then complete their course in a school entirely devoted to medicine, surgery, and the study of disease. The St. George's Hospital Club has its athletic ground of 13½ acres on the Atkinson-Morley Hospital estate at Wimbledon. It provides a football and hockey ground and six tennis courts. Further particulars may be obtained on application to the Acting Dean of the Medical School, Mr. R. R. James, F.R.C.S.

**St. Mary's Hospital Medical School.**—St. Mary's Hospital contains at present 305 beds. Systematic clinical teaching is given daily in the wards and out-patients' department and in the various special departments of the hospital. The Medical School provides for the entire curriculum, and contains well-organised departments for preliminary scientific subjects. The composition fee for the whole curriculum is £200, or £94 10s. for the clinical curriculum only.

**St. Thomas's Hospital.**—The hospital occupies a unique position by the river, and contains 632 beds. The in-patients last year numbered 7,687, whilst the number of attendances as out-patients was 331,278. There are special departments for the treatment of women, children, the eye, ear, nose and throat, skin, and teeth. The tuberculosis department forms a part of the Lambeth scheme for treatment of patients and instruction. The venereal department has been established as part of the L.C.C. scheme. A speech clinic has recently been inaugurated in connection with the children's department. Clinical teaching in the wards, out-patient, and special departments is available every day of the week. All appointments in the hospital are open to students without extra fee. The Students' Club comprises a spacious restaurant, smoking and reading rooms, and there is no occasion for students to leave the hospital buildings during working hours. The annual fees are £25 for the first year (preliminary subjects) and £50 for each subsequent year, covering all tutorial classes, in addition to a fee on entrance. Terms for qualified practitioners may be ascertained from the Medical Secretary to the School, from whom particulars may also be obtained of the five entrance scholarships and a number of prizes and research scholarships offered for competition among the students and newly qualified men.

**University College Hospital.**—Open to men and women students who are able to pursue the whole of their medical studies at University College and University College Hospital. The Medical School comprises the departments of medicine and clinical medicine, surgery and clinical surgery, midwifery and gynæcology, pathology and morbid anatomy and clinical pathology, cardiography, bacteriology, mental physiology and mental diseases, dental surgery, practical pharmacy, and other departments for the study of special diseases, such as those of the eye, skin, ear, and throat, and for instruction in the use of anæsthetics and in electro-therapeutics and the application of the *x*-rays. Whole-time directors of medical and surgical units, with the aid of whole-time assistants, carry out the systematic training of the student in the principles of medicine and surgery, while the practical side is mainly dealt with by the honorary staff of the hospital. Scholarships, exhibitions, and prizes to the value of over £1,000 are offered for competition every year. Composition fees for the courses required by the University of London: For the final M.B., B.S. course, 112 guineas, or in two instalments of 70 and 45 guineas; for the medical education required by the Examining Board in England and the Society of Apothecaries, for the course required for the third examination, 80 guineas, or in two instalments of 50 and 32 guineas. The Dental School, Great Portland Street, W. (formerly the National Dental Hospital), has been recently reorganised and equipped with the most modern appliances for the teaching of dental surgery. The complete dental curriculum can be carried out by students of both sexes at University College and University College Hospital. The fees for the dental curriculum are undergoing revision, but particulars can be obtained from the Sub-Dean for dental students at the Dental School, Great Portland Street.

**Westminster Hospital Medical School.**—Westminster Hospital was the first institution of its kind in London which was founded by the voluntary contributions of the public. There is accommodation for upwards of 200 in-patients. There is a Students' Clubs Union, the subscription for membership of which is included in the general fees. The annual composition fee is 26 guineas. Women students are admitted. Five Entrance Scholarships are offered for competition amongst students entering in October and two for those entering in May.

